# Synthesis of Polyacrylamide Nanomicrospheres Modified with a Reactive Carbamate Surfactant for Efficient Profile Control and Blocking

**DOI:** 10.3390/polym16202884

**Published:** 2024-10-13

**Authors:** Wenwen Yang, Xiaojuan Lai, Lei Wang, Huaqiang Shi, Haibin Li, Jiali Chen, Xin Wen, Yulong Li, Xiaojiang Song, Wenfei Wang

**Affiliations:** 1 College of Chemistry and Chemical Engineering, The Youth Innovation Team of Shaanxi Universities, Shaanxi University of Science & Technology, Xi’an 710021, China; bs230811010@sust.edu.cn (W.Y.); 05wanglei@sust.edu.cn (L.W.); bs210811006@sust.edu.cn (X.W.); 230812107@sust.edu.cn (X.S.); 230811075@sust.edu.cn (W.W.); 2 Shaanxi Agricultural Products Processing Technology Research Institute, Xi’an 710021, China; 3 Oil & Gas Technology Research Institute of Changqing Oilfield Branch Company, PetroChina, Xi’an 710018, China; shqiang_cq@petrochina.com.cn; 4 Xi’an Wonder Energy Chemical Co., Ltd., Xi’an 710018, China; lhb@xawonder.com (H.L.); chenjia6420@163.com (J.C.); 5 Shaanxi Rixin Petrochemical Co., Ltd., Xi’an 710200, China; lyl8705@163.com

**Keywords:** surfactant modified, nanomicrospheres, moderator, blocking rate, recovery rate

## Abstract

Urethane surfactants (REQ) were synthesized with octadecanol ethoxylate (AEO) and isocyanate methacrylate (IEM). Subsequently, reactive-carbamate-surfactant-modified nanomicrospheres (PER) were prepared via two-phase aqueous dispersion polymerization using acrylamide (AM), 2-acrylamido-2-methylpropanesulfonic acid (AMPS) and ethylene glycol dimethacrylate (EGDMA). The microstructures and properties of the nanomicrospheres were characterized and examined via infrared spectroscopy, nano-laser particle size analysis, scanning electron microscopy, and in-house simulated exfoliation experiments. The results showed that the synthesized PER nanomicrospheres had a uniform particle size distribution, with an average size of 336 nm. The thermal decomposition temperature of the nanomicrospheres was 278 °C, and the nanomicrospheres had good thermal stability. At the same time, the nanomicrospheres maintained good swelling properties at mineralization < 10,000 mg/L and temperature < 90 °C. Under the condition of certain permeability, the blocking rate and drag coefficient gradually increased with increasing polymer microsphere concentration. Furthermore, at certain polymer microsphere concentrations, the blocking rate and drag coefficient gradually decreased with increasing core permeability. The experimental results indicate that nanomicrospheres used in the artificial core simulation drive have a better ability to drive oil recovery. Compared with AM microspheres (without REQ modification), nanomicrospheres exert a more considerable effect on recovery improvement. Compared with the water drive stage, the final recovery rate after the drive increases by 23.53%. This improvement is attributed to the unique structural design of the nanorods, which can form a thin film at the oil–water–rock interface and promote oil emulsification and stripping. In conclusion, PER nanomicrospheres can effectively control the fluid dynamics within the reservoir, reduce the loss of oil and gas resources, and improve the economic benefits of oil and gas fields, giving them a good application prospect.

## 1. Introduction

Chinese oil reservoirs are typically formed through terrestrial sedimentation processes, leading to highly heterogeneous geological structures. Consequently, even after extensive water drive operations, approximately 60% of the crude oil remains trapped within the low-permeability regions of these reservoirs. Hence, to improve the overall recovery rate of the oil field, enhancing the recovery rate of crude oil from such low-permeability regions is critical [1,2]. Low-permeability reservoirs encounter several developmental challenges owing to their unique geological characteristics. For instance, these reservoirs often lack natural energy, which results in rapid pressure drops and poses challenges for primary-recovery-rate enhancements. Furthermore, fluid seepage in low-permeability reservoirs follows a non-Darcy seepage pattern [3], governed by complex mechanisms involving pore structures, fluid properties, and their interactions. In such reservoirs, fluid flow behavior deviates considerably from Darcy’s law, primarily due to small pore size, excessively low permeability, strong intermolecular forces acting at phase surfaces, and the Jarman effect. The Jarman effect, in particular, intensifies as such reservoirs deepen, resulting in a decrease in crude oil saturation, an increase in water content, and an enhancement of the capillary drag force. Consequently, numerous oil droplets accumulate at pore throats [4,5,6], causing blockages that obstruct water-driven channels. These blockages severely affect fluid seepage, reduce the effective permeability of reservoirs, and complicate oil extraction. Furthermore, the pore throats of low-permeability reservoirs are typically excessively small and exhibit low permeability, further restricting fluid flow capacity. To overcome these challenges and improve oil recovery from low-permeability reservoirs, new technologies and mobilizers must be developed and implemented. These techniques and driving agents must be designed to improve fluid flow within reservoirs and enhance driving efficiency, ultimately optimizing hydrocarbon recovery [7,8]. Acrylamide (AM)-based microspheres are known for their effectiveness in enhanced oil recovery owing to their tunable particle sizes, excellent swelling characteristics, and strong migration abilities. However, traditional polyacrylamide microspheres are currently inadequate for satisfying the complex requirements of low-permeability reservoirs, primarily owing to the harsh field conditions, increased structural heterogeneity, and growing numbers of these reservoirs. Furthermore, single-function drive modifiers are unable to substantially enhance reservoir recovery, particularly in reservoirs with multiple major channels and fractures. Their limited ability to adjust flow and effectively manage fluid short-flow and scuttling phenomena [9,10] can considerably reduce the final recovery efficiencies of reservoirs. Thus, to address the challenges encountered during low-permeability reservoir development, innovative driving agents must be developed urgently. These agents must be endowed with water-absorbing and swelling functionalities to effectively seal fine pores and throats within reservoirs, along with adequate deformation capacity to penetrate these orifices. When traversing a formation, driving agents must be uniformly distributed across the three-phase oil–water–rock interface, generating strong adsorption and stripping forces. These forces can not only help emulsify and strip crude oil but also considerably improve the overall recovery rate of the reservoir. In the context of oil repulsion mechanisms, most studies on the functionalization of polymer microspheres have predominantly focused on enhancing their blocking strengths [11,12,13,14,15]. There are few studies on improving the interfacial activity and structural separation pressure of polymers by modifying them with surfactants.

The surfactants can be divided into conventional surfactants and reactive surfactants according to whether the structure contains reactive groups. The conventional surfactant molecules have one hydrophilic group and one hydrophobic group. Reactive surfactant molecules incorporate polymerizable reactive groups, primarily unsaturated alkenes, within the conventional surfactant molecular structure; reactive surfactants not only have the characteristics of conventional surfactants but also can copolymerize with unsaturated monomers under high temperatures or in the presence of an initiator. Residual surfactants may cause environmental problems, and augmenting the presence of free surfactants in the final product may have some negative effects on product performance. The emergence of reactive surfactants can solve these problems in the application of conventional surfactants. Reactive surfactants are copolymerized directly into the main polymer chain through covalent bonding during polymerization. Reactive surfactants can address the disadvantages of many conventional surfactants and can be used in various fields, such as construction, paper manufacturing, leather production, industrial coating, and biological medicine. However, the use of modified polyacrylamides with reactive surfactant functionalities in oil recovery applications still remains relatively unexplored; in particular, the effect of using surfactant-modified polyacrylamides on the rheological properties of polymer waterproofing fluids has not been systematically studied [16,17,18]. Based on this background, a surface-active functional monomer named REQ was designed and synthesized to meet the practical application requirements of reducing particle size and increasing hydrophilicity, as well as increasing the structural separation pressure to improve the emulsion stripping of crude oil. The monomer REQ was subsequently copolymerized with AM, 2-acrylamido-2-methylpropanesulfonic acid (AMPS), and ethylene glycol dimethacrylate (EGDMA) to prepare nanomicrospheres named PER by radical polymerization. Subsequently, the properties of these nanomicrospheres were evaluated based on their microstructures, thermal stability, swelling properties, temperature and salt resistance, and in-house physical simulation of exclusion experiments. Results show that the PER nanomicrospheres have a narrow particle size distribution, excellent swelling properties, and high blockage and recovery rates and can effectively control the fluid dynamics within the reservoir. In addition, they help to reduce the loss of oil and gas resources, improve economic efficiency, and provide considerable strategic value in advancing the development of petroleum engineering technology.

## 2. Materials and Methods

### 2.1. Materials

AM and AMPS (both of analytical grade) were obtained from Tianjin CITIC Kaitei Chemical Co. (Tianjin, China). In addition, octadecanol ethoxylate (AEO), polyvinylpyrrolidone, isocyanate methacrylate (IEM), and acetone (all of analytical grade) were obtained from Shanghai McLean Biochemical Technology Co. (Shanghai, China). Azodiisobutylamidine hydrochloride (V50), ammonium persulfate, sodium chloride, potassium chloride, magnesium chloride, calcium chloride, sodium hydroxide, and sodium formate were provided by Sinopharm Chemical Reagent Co. (Shanghai, China).

### 2.2. Equipment

The following equipment was used. The reaction process was monitored using an NTC temperature sensor from Guangdong Shengze Technology Co. (Chaozhou, China) Scanning electron microscopy (SEM) was performed using a Quattro scanning electron microscope (Quattro SEM) from Beijing Opto Optical Technology Co. (Beijing, China). A thermogravimetric analyzer (TGA550) from Cloud Spectrum Instruments (Shanghai) Co Ltd. (Shanghai, China) was used for thermal analysis. A Fourier-transform infrared (FTIR) spectrometer (Shimadzu, AIM-8800, Tokyo, Japan) was used for structural analysis. A multifunctional core filtration loss substitution device (DLS-5) from Haian Petroleum Research Instruments Co. (Hiana, China) was used for filtration studies. A laser particle sizer (Betterize2000) from Laihen Technology (Beijing) Co. Ltd. (Beijing, China) was used for particle size analysis. Finally, an electrothermal constant temperature digital display water bath (KHW-D-1) from Beijing Capri Medical Instruments Co. Ltd. (Beijing, China) and an electromagnetic stirrer (B11-1) from Zhejiang Nade Technology Co. Ltd. (Hangzhou, China) were used for sample preparation.

### 2.3. Synthesis of the REQ Monomer and PER Nanomicrospheres

**Figure 1:**
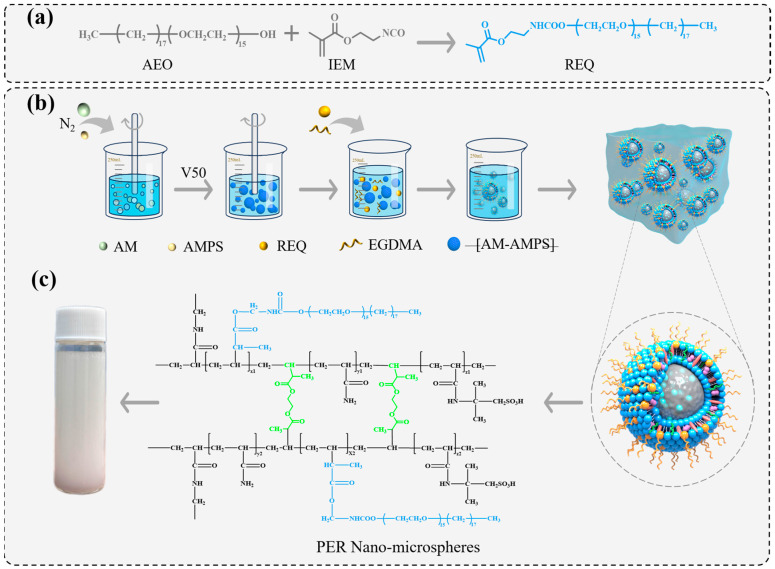
(**a**) Synthetic route for the REQ functional monomer. (**b**) Synthetic route for the PER nanomicrospheres. (**c**) Molecular formula for PER nanomicrospheres.

Initially, AEO was dried in a vacuum oven at 50 °C for 10 h to eliminate residual moisture. Subsequently, AEO, IEM, and acetone were mixed in a dry three-necked flask in a ratio of m(AEO):m(IEM):m(acetone) = 1:1.1:10 This mixture was subjected to electric stirring at 40 °C for 4 h. The resulting product was washed, precipitated, and filtered using ether under reduced pressure. The obtained product was then dried under vacuum for 24 h at 25 °C, yielding the surface-active functional monomer REQ, as indicated in the reaction formula depicted in Figure 1a.

For the synthesis of PER nanomicrospheres, two-phase aqueous dispersion polymerization was adopted. Herein, a monomer composition mass ratio of m(AM):m(H_2_O):m(AMPS):m(REQ):m(EGDMA) = 20:42:20:17:0.3 was used. In particular, AM was dissolved in distilled water, following which AMPS and REQ were added to the solution and homogeneously mixed to produce the monomer phase. The pH of this phase was adjusted to a value of 5.5 while maintaining its temperature within 25 °C. Subsequently, a saturated brine phase was prepared using distilled water mixed with predefined proportions of ammonium sulfate, sodium chloride, and sodium formate. This brine phase was then added to an open glass reactor and subjected to inert gas deoxygenation. After 30 min, the monomer phase and polyvinylpyrrolidone were added to the system, and an initiator solution (0.2% wt. V50) was introduced through a catheter. The injection rate of the microsampler was set to 0.5 mL/h, while the temperature of the system was adjusted to 40 °C. Once the color of the emulsion changed from white to off-white (indicating phase transition), the temperature of the system was increased to 70 °C. The reaction was allowed to continue for 1 h until the emulsion turned milky white, yielding PER nanomicrospheres. The primary reaction mechanisms are illustrated in Figure 1b, while Figure 1c presents the molecular formula for the PER nanomicrospheres.

### 2.4. Characterization and Performance Testing

#### 2.4.1. Characterization

Structural characterizations of dry powders of the monomer and PER nanomicrospheres were performed via infrared spectroscopy with a potassium bromide press. In addition, the surface morphology of the freeze-dried nanomicrospheres was imaged using a Quattro scanning electron microscope from Beijing OPTO Optical Technology Co. (Beijing, China). Before imaging, the sample surfaces were coated with a thin layer of gold [19]. Next, the heat resistance of the viscoelastic nanomicrospheres was assessed using a thermogravimetric analyzer (TGA550) from Shenzhen Teli Instrumentation Co, Ltd, (Guangdong, China) under a nitrogen atmosphere. During this analysis, the temperature was increased at a rate of 10 °C/min from 20 °C to 600 °C.

The average particle size and distribution of the dry powder of the PER nanomicrospheres were quantified in anhydrous ethanol using a laser-diffraction-based dry laser particle sizer (Bettersize2000) from Shenzhen Teli Instrumentation Co, Ltd., (Shenzhen, China) [20]. The average particle size and distribution of the nanomicrosphere emulsions were evaluated in a 0.3 wt% aqueous nanomicrosphere solution. Before particle size measurements at 25 °C, all dispersions were subjected to mild ultrasonication to ensure proper dispersion. The particle concentration in the dispersions was 0.05%, and the samples were aged at 25 °C for 1, 3, and 5 d.

#### 2.4.2. Performance Testing

(1) Salt resistance: For determining salt resistance, 1000-, 2000-, 5000-, 10,000-, and 20,000-mg/L brine solutions of sodium chloride, potassium chloride, magnesium chloride hexahydrate, and calcium chloride were prepared according to a mass ratio of 10:5:1:1.5. Subsequently, an appropriate amount of the PER nanomicrospheres was uniformly dispersed in the brine solutions to create dispersion systems of 1000 mg/L microspheres. The nanomicrospheres were tested for particle size and their swelling multiplicity Q at 50 °C.

(2) Dissolution multiplier: A 1000-mg/L nanomicrosphere solution was prepared using distilled water and stirred for 2 h to avoid the sedimentation and accumulation of nanomicrosphere particles. The solution was then transferred to a 50 °C oven for storage. Microsphere particle size analysis and microscopic morphology observation were conducted on the 1st, 3rd, 5th, 7th, 9th, 10th, and 12th days. The dissolution multiplier of the nanomicrospheres, *Q*, was calculated *Q*(1). Subsequently, the solution was then transferred to an oven for storage at 50 °C.

(1) $$Q=\left(D_{2}-D_{1}\right)/D_{1}$$

where *Q* represents the dissolution multiplier, *D*_1_ represents the particle size of the nanomicrospheres before swelling (nm), and *D*_2_ represents the particle size of the nanomicrospheres after swelling (nm).

(3) Temperature resistance: Based on the aforementioned dissolution rate test procedure and requirements, nanomicrosphere particle size analysis was conducted at different temperatures (50 °C, 70 °C, 90 °C, and 110 °C), and *Q* was calculated.

#### 2.4.3. Blocking Test

In this experiment, a pressure sensor was installed on the core to observe the changes in the pressure gradient during the injection of the nanomicrospheres and to examine their blocking ability. The experimental steps are described below.

(1) A square-bar artificial core model (dimensions: 4.5 cm × 4.5 cm × 30 cm) with three pressure measurement points (the core injection port is the first pressure measurement point, the middle of the core is the second pressure test point, and the end of the core is the third pressure test point) was used in the experiment. Artificial cores with permeabilities of 800 × 10^−3^ µm^2^, 2000 × 10^−3^ µm^2^, and 4000 × 10^−3^ µm^2^ were used, and the experimental temperature was 70 °C. The core was driven by water at a driving rate of 1 mL/min until the pressure became stable.

(2) Working solutions of nanomicrospheres with concentrations of 2000, 3000, and 4000 mg/L were prepared and placed in the container.

(3) The nanomicrosphere solution (1 PV) was injected into the core, after which the core was closed and saved to enable microsphere aging in the core for 7 d under a stable temperature of 70 °C.

(4) After 7 d, 1.5 cm of the injected end of the core was cut off, and subsequent water drive was conducted at a speed of 1 m/min. The pressure changes at each pressure point were recorded in real time, and the blocking rate, resistance coefficient, and residual resistance coefficient were calculated when the injection pressure stabilized, marking the end of the experiment.

Following this, its permeability was calculated using Darcy’s law (2) once the injection pressure stabilized:

(2) $$K_{1}=\frac{10^{6}Q\mu L}{A\Delta P}$$

where *K*_1_ represents the hydrometric permeability of the homogeneous core (10^−3^ μm^2^), *Q* represents the fluid flow rate (mL/min), *μ* represents the viscosity of water (mPa·s), *L* represents the length of the core (cm), *A* represents the cross-sectional area of the core (cm^2^), and ∆*P* represents the pressure difference across the core (MPa).

Once the water drive pressure stabilized under the given flow rate, the nanomicrosphere dispersion was injected into the core with an injection volume of 10 times the pore volume. The pressure at each measurement point was recorded, and the permeability of the core after nanomicrosphere injection (*K*_2_) was measured. The blocking rate (*η*) was then calculated as follows:

(3) $$\eta =\frac{K_{1}-K_{2}}{K_{1}}\times 100\%.$$

The drag coefficient is calculated as shown in Equation (4):

(4) $$R_{f}=\frac{\Delta P_{3}}{\Delta P_{1}},$$

where *Rf* represents the drag coefficient of the core, ∆*P*1 represents the differential pressure between the two ends of the core after the front water drive (MPa), and ∆*P*3 represents the differential pressure at the ends of the core after subsequent water drives (MPa).

The residual drag coefficient is calculated as shown in Equation (5):

(5) $$R_{\mathrm{ff}}=\frac{\Delta P_{1}}{\Delta P_{2}}$$

where *R*_ff_ represents the residual drag coefficient of the core, ∆*P*_1_ represents the differential pressure between the two ends of the core after the front water drive (MPa), and ∆*P*_2_ represents the differential pressure at the ends of the nanomicrosphere drives (MPa).

#### 2.4.4. Oil Drive and Recovery Experiment

In the oil drive and recovery experiment, microsphere solvents with concentrations of 2000, 3000, and 4000 mg/L were selected for reservoirs with a permeability of 2000 × 10^−3^ µm^2^ to improve recovery, and the results were compared with those of AM microsphere (without REQ modification) oil drive and recovery experiments.

(1) The specific steps of the experiment are as follows:

(2) The core was vacuum-saturated with formation water at a reservoir temperature of 70 °C, and the pore volume was calculated; the oil saturation was determined.

(3) In the water drive stage, the core was water-driven to 90% water content to achieve water drive recovery.

(4) In the injection stage, polymer nanomicrospheres were injected at 0.3 PV, followed by a water drive for 5 min and slow expansion for 7 d.

(5) After 7 d, a subsequent water drive was performed to reach 98% water content, and the final recovery was calculated.

The experimental injection rate was 1 mL/min, and the time interval was 10 min. During the experiment, the injection pressure, the amount of discharged liquid, and the amount of discharged oil were recorded. At the end of the experiment, the water content and recovery were calculated, and the relevant characteristic curves were plotted.

## 3. Results and Discussion

### 3.1. Characterization of Nanomicrospheres

#### 3.1.1. Changes in Temperature Rise during Polymerisation

**Figure 2:**
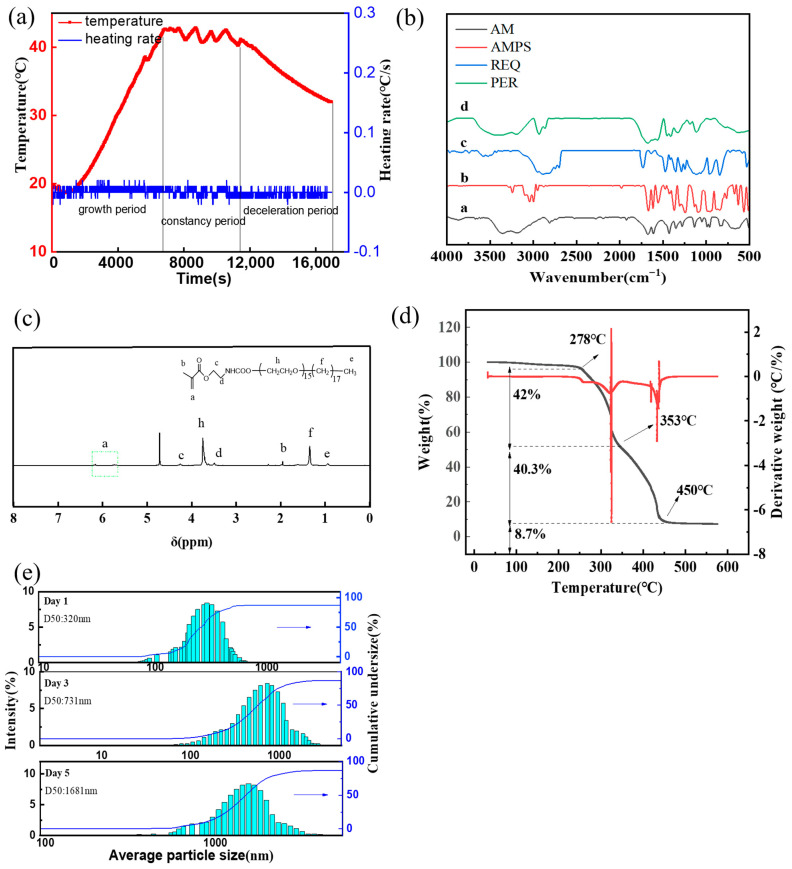
(**a**) Diagram of polymerization heating process. (**b**) FTIR spectra of AM (curve a), AMPS (curve b), REQ monomer (curve c), and PER nanomicrospheres (curve d). (**c**) NMR of the monomer REQ. (**d**) PER thermal stability test. (**e**) Particle size testing of PER nanomicrospheres with different dissolution times.

An NTC temperature sensor was used to accurately track the temperature fluctuations during the nanomicrosphere polymerization reaction, as illustrated in Figure 2a. The reaction proceeds through three distinct phases: acceleration, stabilization, and deceleration.

In the acceleration phase, the polymerization reaction is catalyzed by the introduction of initiators or short-chain free radicals, resulting in a steady increase in the polymerization rate. This phase is also the nucleation stage, where the formation of new particles occurs. Subsequently, the reaction progresses into the stabilization phase, a critical period for polymer chain elongation. During this phase, the reaction remains highly active, necessitating the maintenance of the reaction temperature within an optimal range to facilitate the growth of the polymer chains. The final phase is the deceleration stage. During this period, the remaining monomers continue to react, although at a considerably reduced rate. The reaction concludes when all active monomers have been fully converted into polymers. Following the completion of these three phases, the nucleation reaction is finalized, and the system begins to cool down. The resultant product is a milky white emulsion of polymer nanomicrospheres, indicating that the polymerization reaction adheres to the typical process of emulsion polymerization [21].

#### 3.1.2. Fourier Transform Infrared (FTIR) Spectroscopy

Figure 2b depicts the FTIR spectra of AM, AMPS, the REQ monomer, and PER nanomicrospheres. In this figure, curve a exhibits several important absorption peaks. For instance, peaks corresponding to the stretching vibrations of the N–H bonds in the amino group (–NH_2_) are located at 3355 and 3195 cm^−1^. The stretching vibration of the carbonyl group in the amide moiety (C=O) presents a strong absorption peak at 1678 cm^−1^. Furthermore, the characteristic absorption peak of the stretching vibration of the C=C double bond is observed at 1608 cm^−1^. Furthermore, the characteristic absorption peaks of the out-of-plane bending vibrations of the unsaturated double bond are observed at 971 and 820 cm^−1^. In the case of curve b, the absorption peak observed at 3038 cm^−1^ corresponds to the stretching vibration of the C–H bond in the unsaturated C=C double bond. Moreover, the characteristic absorption peaks corresponding to the out-of-plane bending vibrations of the unsaturated double bond are observed at 950 cm^−1^ and 837 cm^−1^. Furthermore, the antisymmetric stretching vibration of the sulfate group (O=S=O) gives rise to two peaks at 1238 cm^−1^ and 1095 cm^−1^. Curve c indicates the absence of the characteristic peak of isocyanate (–NCO) at 2277 cm^−1^, indicating its complete consumption during the reaction. Furthermore, the appearance of the characteristic peak of stearyl alcohol-polyoxyethylene ether (C–O–C) at 1103 cm^−1^ confirms the successful synthesis of the functional monomer REQ. Curve d reveals the stretching vibration absorption peaks of the N–H bonds in the amide group at 3354.06 cm^−1^ and 3201.69 cm^−1^. Moreover, the stretching vibration absorption peaks corresponding to the C–H bonds in methyl (–CH_3_) and methylene (–CH_2_) are observed at 2929.74 cm^−1^. Furthermore, the stretching vibration absorption peak of the carbonyl group (C=O) in the amide moiety is observed at 1658.71 cm^−1^. The in-plane bending vibration absorption peaks of the methyl and methylene groups are apparent at 1452.33 cm^−1^. The bending vibration absorption peak corresponding to the C–O bond is observed at 1319.25 cm^−1^, while its telescopic vibration absorption peak is observed at 1125.52 cm^−1^. Additionally, the characteristic absorption peak corresponding to the C–S bond in the sulfonic acid moiety indicates that the desired molecular structure has been successfully achieved.

#### 3.1.3. NMR of the Monomer REQ

Figure 2c shows the 1H-NMR spectra of REQ. The various chemical-shift (δ) values in the 1H-NMR spectrum shown in Figure 2c could be assigned as follows: 5.55–6.15 [aH, CH_2_=C(CH_3_)–], 1.81 [bH, CH_2_=C(CH_3_)–], 4.20–4.35 (cH, –COOCH_2_CH_2_–), 3.30–3.75 (hH, –OCH_2_CH_2_–OOC–), 1.26–1.56 [fH, –(CH_2_)_17_–CH_3_], and 0.85–1.10 [eH, –(CH_2_)_17_–CH_3_]. These data confirmed that the products were double-bond-containing carbamate surfactants.

#### 3.1.4. Thermal Stability Test

As depicted in Figure 2d, the thermal decomposition of the PER nanomicrospheres occurs in three distinct stages. In the first stage (at ~278 °C), the sulfonic acid and amide groups within the polymers exhibit strong hydrophilicity. As the temperature increases, water from these groups volatilizes, causing the partial decomposition of the amide groups and a consequent loss of weight. The second stage (278 °C–353 °C) involves the imidation reaction of the amide groups and the thermal decomposition of the hydrophobic side chains. During this period, any unreacted residual monomers are consumed. In the third stage (353 °C–450 °C), the primary decomposition of organic matter within the polymer ensues. For instance, the primary and branched chains of the polymer begin to disintegrate, while impurities generated via side reactions decompose. When the thermal decomposition temperature reaches 450 °C, approximately 8.79% of the PER nanomicrospheres remain. Notably, the functional monomer REQ strengthens intermolecular bonding, effectively improving the thermal stability of the nanomicrospheres.

#### 3.1.5. Laser Particle Size Testing

The particle sizes of the PER nanomicrospheres were measured at various aging times using a nanoparticle size analyzer. As depicted in Figure 2e, the initial particle sizes of the PER nanomicrospheres are normally distributed between 295 and 469 nm, with an average size of 336 nm. This distribution indicates that the nanomicrospheres are uniformly dispersed, with a narrow size range. After 3 d of aging, the particle sizes of the PER nanomicrospheres appear to be normally distributed between 684 and 933 nm, with an average size of 785 nm. This indicates that the sizes of the PER nanomicrospheres increase to more than three times their original sizes after swelling. After 5 d of aging, the particle sizes of the PER nanomicrospheres are normally distributed between 971 and 1341 nm, with an average particle size of 1035 nm. During this aging period, a slight deformation of the spherical surface of PER nanomicrospheres and a breakage of some of the long molecular chains occur. If aging continues beyond this point, the PER nanomicrospheres are anticipated to transform from spherical particles into irregular, shuttle-like molecular fragments, with the solution viscosity approaching that of water.

#### 3.1.6. Environmental SEM Microscopy Testing

**Figure 3:**
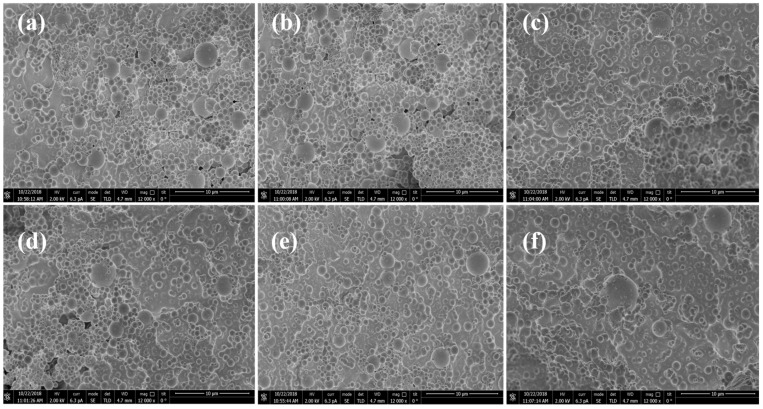
Scanning electron microscopy of nanomicrospheres PER under different conditions: (**a**) dissolved in pure water for 1 d, (**b**) dissolved in pure water for 3 d, (**c**) dissolved in pure water for 5 d, (**d**) dissolved in saline for 1 d, (**e**) dissolved in saline for 3 d, and (**f**) dissolved in saline for 5 d.

As illustrated in Figure 3a, when pure water is used as the solvent, as illustrated in Figure 3a, the surface morphology of the nanomicrospheres after 1 d of swelling in pure water reveals a considerably uniform particle size distribution, with nanomicrospheres that are full and regular in shape. The degree of branching of the long polymer chains inside the nanomicrospheres is moderate after 3 d of swelling (Figure 3b), maintaining an orderly arrangement even at the microscopic scale. This ordered structure endows the nanomicrospheres with ideal mechanical properties and stability. By the fifth day of swelling (Figure 3c), slight deformations are observed on the surface of some nanomicrospheres, indicating rapid volume expansion and strong water absorption. As illustrated in Figure 3d, when saline water is used as the solvent, as depicted in Figure 3d, the amide groups in the nanomicrospheres undergo hydrolysis, gradually transforming into carboxyl groups. The electrostatic repulsion between the carboxyl groups causes the further stretching of the polymer chains within the nanomicrospheres, leading to volume expansion while maintaining a full and regular appearance. By the third day of swelling in saline water (Figure 3e), more water molecules penetrate the nanomicrospheres and subsequently diffuse into their interiors. Partial stacking interactions may occur between the polymer chains inside the nanomicrospheres, possibly due to intensified thermal motion resulting in changes in intermolecular forces. As a result of these stacking interactions, the nanomicrospheres rapidly reach a swelling equilibrium. By the fifth day (Figure 3f), the surface morphology of the nanomicrospheres undergoes considerable changes, with an even more pronounced water absorption and expansion. The interactions with water molecules are notably enhanced. The improvement in the dissolution and swelling behavior of the nanomicrospheres may improve their water absorption characteristics and swelling capacity. These changes at the microscale level have substantial implications for the overall properties of the nanomicrospheres. This indicates that a detailed understanding of the swelling mechanism in different solvents could result in the development of nanomicrospheres with tailored properties for specific applications [22].

### 3.2. PER Nanomicrospheres Performance Testing

#### 3.2.1. Salt and Temperature Resistance Testing of PER Nanomicrospheres

**Figure 4:**
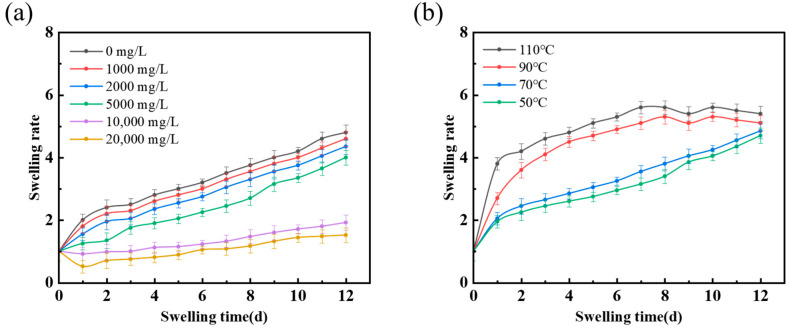
Salt and temperature resistance of PER nanomicrospheres. (**a**) Comparison of salt resistance of nanomicrospheres at different salt concentrations. (**b**) Comparison of the temperature resistance of nanomicrospheres at different temperatures.

As illustrated in Figure 4a, the influence of salt concentration on the swelling behavior of nanomicrospheres is primarily manifested in two facets. First, the interaction between ions in the salt and the polymer chains reduces the mobility of the polymer chain segments, particularly at high salt concentrations (>10,000 mg/L). This reduction in mobility decreases the swelling capability of the nanomicrospheres in the solvent. Second, the presence of salt hinders the solvent from penetrating the polymer structure via osmotic action, thereby altering the swelling equilibrium and causing a substantial reduction in the degree of swelling of the nanomicrospheres. Experimental observations indicate that as the salt concentration in the system increases, the swelling rate of the nanomicrospheres is reduced, with a swelling ratio that is notably lower than that observed in a distilled water system. This trend becomes more pronounced when the salt concentration exceeds 10,000 mg/L, indicating a more pronounced decline in the swelling ratio of the nanomicrospheres.

The role of temperature on the swelling performance of the nanomicrospheres is primarily observed in two key aspects. First, increased temperature enhances the swelling of the nanomicrospheres by increasing the thermal energy of the polymer chains, which in turn promotes chain motion and extension. Consequently, the contact area with the solvent increases, which facilitates the adsorption of solvent molecules and thereby increases the degree of swelling. Second, the swelling equilibrium state is influenced by temperature. Within an appropriate temperature range, an increase in temperature is conducive to an increase in the degree of swelling. However, excessively high temperatures (>90 °C) can cause structural damage or the degradation of the polymer chains, which paradoxically reduces swelling performance.

As illustrated in Figure 4b, under identical swelling conditions, the swelling ratio of the nanomicrospheres notably increases with increasing temperature under identical swelling conditions. However, when the temperature exceeds 90 °C, a decrease in the swelling ratio is observed on the ninth day, primarily caused by the rupture of some nanomicrospheres due to excessive swelling. This indicates that while temperature considerably promotes the swelling of the nanomicrospheres, there is an optimal temperature range that must be maintained to achieve the maximum swelling efficiency without compromising the structural integrity of the nanomicrospheres. Beyond this optimal range, the detrimental effects of high temperatures on the polymer chains can lead to a decline in swelling performance and potential structural damage.

#### 3.2.2. Experiments on the Blocking Properties of Nanomicrospheres in Cores with Different Permeabilities

To investigate the blocking conditions of different nanomicrospheres for various permeability reservoirs, the above experimental method was used. Nanomicrosphere blocking experiments were conducted in a 2000 mg/L nanomicrosphere solution using artificial cores with permeabilities of 800 × 10^−3^ µm^2^, 2000 × 10^−3^ µm^2^, and 4000 × 10^−3^ µm^2^. The results of the experimental data are presented in Table 1.

**Figure 5:**
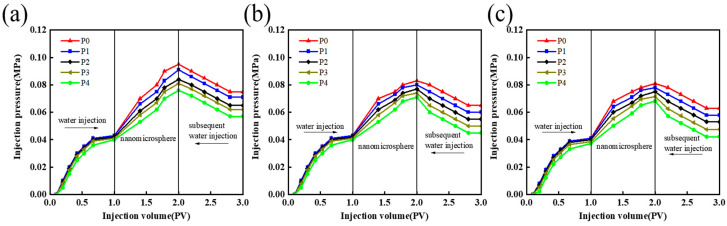
Pressure characteristic curve of each pressure point under different permeability. (**a**) Core permeability of 800 × 10^−3^ µm^2^. (**b**) Core permeability of 2000 × 10^−3^ µm^2^. (**c**) Core permeability of 4000 × 10^−3^ µm^2^.

As can be seen from Table 1 and Figure 5, for the three different permeability cores with different permeabilities, the resistance coefficient and residual resistance coefficient are lower in the case of higher permeability after nanomicrosphere injection. When the permeability is 800 × 10^−3^ µm^2^ (Figure 5a), the drag coefficient and residual drag coefficient reach 4.51 and 7.82, respectively, and the blocking rate reaches 86.07%. When the permeability is 2000 × 10^−3^ µm^2^ (Figure 5b), the drag coefficient and residual drag coefficient decrease to 3.47 and 6.01, respectively, and the blocking rate reaches 79.13 %. When the permeability is 4000 × 10^−3^ µm^2^ (Figure 5c), the residual resistance coefficient and residual resistance coefficient are only 2.55 and 4.53, respectively, and the blocking rate is 68.21%. Under different core permeabilities, the pressure gradient increases with increasing proximity to the injection end. Owing to the “end face effect,” the pressure gradient value and blocking rate in the P0–P1 interval are higher than those of other intervals. In the subsequent water drive stage, compared with other intervals, the pressure gradient in the P0–P1 interval is also higher than in other intervals; moreover, the blocking rate is also higher in this interval, indicating that the amount of microsphere retention in this interval is larger. This is because the higher the permeability, the larger the pore structure inside the core, allowing the nanomicrospheres to more easily enter the rock interior. Because it is difficult for these microspheres to form an effective aggregate seal at the pore throat, their retention in the formation is reduced, leading to a lower sealing rate. Thus, for reservoirs with high permeability, the injection performance of nanomicrospheres demonstrated better injection performance but a relatively low blocking effect [23,24].

#### 3.2.3. Experiments on the Blocking Properties of Nanomicrospheres in Cores for Different Concentrations

To investigate the blockage of low permeability reservoirs for different concentrations of nanomicrospheres, blockage experiments were conducted on an 800 × 10^−3^ µm^2^ artificial core using 2000, 3000, and 4000 mg/L of nanomicrospheres according to the aforementioned experimental method. The results are presented in Table 2.

**Figure 6:**
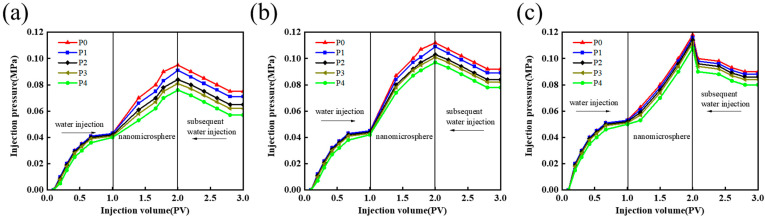
Pressure characteristic curve of each pressure point under different concentrations. (**a**) 2000 mg/L PER nanomicrosphere dispersion. (**b**) 3000 mg/L PER nanomicrosphere dispersion. (**c**) 4000 mg/L PER nanomicrosphere dispersion.

For an 800 × 10^−3^ µm^2^ permeability core, the resistance coefficient and residual resistance coefficient for three concentrations of nanomicrosphere solvent injection increase with the increasing concentration, as shown in Table 2 and Figure 6. When the nanomicrosphere concentration is 2000 mg/L (Figure 6a), the drag coefficient and residual drag coefficient reach 4.33 and 8.70, respectively, and the blocking rate reaches 83.03%. When the microsphere concentration is 3000 mg/L (Figure 6b), the drag coefficient and the residual drag coefficient increase to 5.60 and 8.23, respectively, and the blocking rate is 85.48%. When the nanomicrosphere concentration is 4000 mg/L (Figure 6c), the resistance coefficient and residual resistance coefficient are 5.71 and 9.75, respectively, and the blocking rate is 90.32%. This is because a higher concentration leads to a greater number of nanomicrospheres in the unit volume, making it easier for the nanomicrospheres to adhere and bridge at the microscopic pore throat to form a blockage. This results in the higher retention of the formation, an increased injection pressure, and therefore a higher blockage rate. Thus, for reservoirs with the same permeability, the blockage effect of the nanomicrospheres at high concentrations can be improved [25].

When analyzing the blocking effect in the second half of the core, the nanomicrosphere system with a concentration of 4000 mg/L exhibited a stronger blocking ability compared with the systems with 2000 and 3000 mg/L (Figure 6). This is because a higher concentration resulted in more nanomicrospheres being retained in the core, and these nanomicrospheres could undergo several cycles of transport, sealing, and re-transport in the core under subsequent water drive, thereby accumulating in the second half of the core and forming an effective blockage. In contrast, at lower concentrations, the number of nanomicrospheres decreased, leading to weakened adhesion and agglomeration effects, making it difficult to form effective stacking and bridging blockage at the pore throat. Thus, nanomicrospheres were easily discharged from the core along with the dominant channels, which reduced the blocking rate.

### 3.3. Experiments on Oil Drive and Recovery

This subsection describes the examination of the actual oil drive and recovery performance of nanomicrospheres. According to the aforementioned experimental method, the configured nanomicrosphere solutions of different concentrations were injected into the experimental model device, dissolved at 70 °C for 24 h, and subsequently subjected to water drive. Records were kept during the experiment, and the obtained experimental data and results were plotted as experimental dynamic curves.

#### 3.3.1. Experiment on Oil Recovery and Enhancement by a Microsphere System at 2000 mg/L

**Figure 7:**
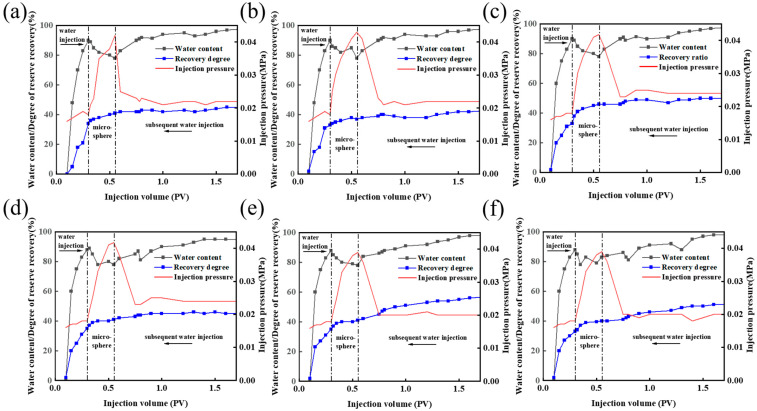
Dynamic characteristics of polymer microsphere oil repulsion experiments at different concentrations. (**a**) PER nanomicrospheres at a concentration of 2000 mg/L. (**b**) AM microspheres at a concentration of 2000 mg/L. (**c**) PER nanomicrospheres at a concentration of 3000 mg/L. (**d**) AM microspheres at a concentration of 3000 mg/L. (**e**) PER nanomicrospheres at a concentration of 4000 mg/L (**f**) AM microspheres at a concentration of 4000 mg/L.

As shown in Figure 7a,b, at a concentration of 2000 mg/L, no difference is observed in the characteristic curves and recovery rates between the two microsphere systems in the pre-injection water drive. After the injection of polymer microspheres, the pressure and recovery of the core conditioned with PER nanomicrospheres were better than those of the core conditioned with AM microspheres. Moreover, the final recovery increased by 13.12% compared with that in the pre-water drive stage, while the recovery of the core conditioned with AM microspheres increased by 10.09%. The core recovery of the PER nanomicrosphere system increased by 3.03% compared with the AM microspheres. Further analysis revealed that the injection pressure tended to flatten in the water drive stage and that the water content rapidly increased in the early stage of the water drive, indicating the presence of a hypertonic dominant channel in the core. After the injection of the microspheres, the dominant flux channel in the hypertonic layer was blocked, the injection pressure increased, and the water content showed a fluctuating downward trend; moreover, a gradual increase in the recovery rate was observed at this stage. With the continuation of the subsequent water drive, the injection pressure began to decrease and stabilize; however, it remained higher than the injection pressure in the pre-water drive. As the subsequent water drive continued, the injection pressure started to decrease and stabilize, but it remained higher than the injection pressure in the pre-water drive. The water content gradually increased to the maximum value, and the recovery rate increased considerably compared with that of the pre-water drive stage, indicating that the PER nanomicrospheres exhibit considerably high performance in terms of oil repulsion and recovery.

#### 3.3.2. Experiment on Oil Recovery and Enhancement by a Microsphere System at 3000 mg/L

As shown in Figure 7c,d, similar to the case of 2000 mg/L, no difference is observed in the characteristic curves and recovery rates between the two microsphere systems in the pre-injection water drive. After the injection of polymer microspheres, the pressure and recovery of the core conditioned with PER nanomicrospheres exhibited a considerable increase compared with those of the core conditioned with AM microspheres, and the final recovery increased by 18.46% relative to that of the pre-water drive stage, while the recovery of the core conditioned with AM microspheres increased by 12.28%. The core recovery of PER nanomicrospheres system increased by 6.18% compared with AM microspheres, indicating that PER nanomicrospheres exert a more pronounced effect of water reduction and oil enhancement and exhibit better performance in terms of oil recovery [26,27].

#### 3.3.3. Experiment on Oil Recovery and Enhancement by a Microsphere System at 4000 mg/L

As shown in Figure 7e,f, at the concentration of 4000 mg/L, no difference is observed in the characteristic curves and recovery rates of the two microsphere systems in the pre-water drive. When the polymer microsphere drive was started, the pressure and recovery of the core conditioned with PER nanomicrospheres were higher than those of the core conditioned with AM microspheres. Moreover, the final recovery increased by 23.53% relative to that of the pre-water drive stage, while the recovery of the core conditioned with AM microspheres increased by 15.70%. The core recovery of the PER nanomicrosphere system increased by 7.83% compared with AM microspheres.

A comparison of the aforementioned data for concentrations of 2000, 3000, and 4000 mg/L of PER nanomicrospheres and AM microspheres shows that the final recovery rate and the increase in the recovery rate increase with increasing concentration for PER nanomicrospheres and AM microspheres. This indicates that for the microsphere driving system, the effects of water reduction and oil increase will be more pronounced at higher concentration. Under the same concentration, the recovery rate and the increase in recovery rate of PER nanomicrospheres system have better performance compared with that of AM microspheres, indicating that the nanomicrosphere system has better performance driving oil and improving oil recovery in reservoirs with enhanced oil recovery operation [28,29,30].

### 3.4. PER Nanomicrospheres Enhanced Oil Recovery Analysis

**Figure 8:**
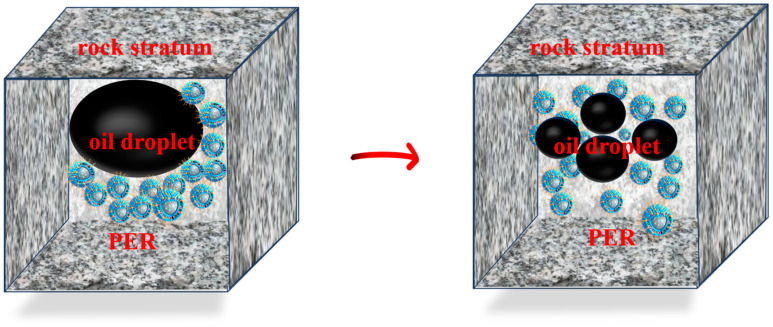
PER nanomicrospheres oil repellent diagram.

In the realm of enhanced oil recovery methods, surface-active modified nanomicrospheres, such as PER, play crucial roles in solid–liquid systems. As shown in Figure 8, when these nanomicrospheres accumulate at the oil–water interface, they use surfactant properties to form a stable film. This film acts as a lubricating barrier, significantly reducing the contact angle between oil droplets and the rock surface, thereby decreasing the adhesion force between oil droplets and rock. The oil droplets, which were previously tightly adhered, become loosened and are more easily dislodged or peeled off from the rock surface, significantly enhancing oil mobility. This process not only facilitates the migration of oil droplets but also optimizes the distribution of the oil displacement agent within the rock pores, thereby improving the efficiency of oil displacement. Furthermore, the addition of nanomicrospheres can improve the rheological properties of the fluid, reducing the viscosity of crude oil, further increasing the recovery rate of the reservoirs and significantly contributing to the economic benefits of oil fields [31,32,33,34,35].

## 4. Conclusions

Surfactant-modified nanomicrospheres were synthesized using a two-phase water dispersion polymerization method, with AM and AMPS used to construct the molecular skeleton. REQ was used as the surfactant-modified monomer, while EGDMA served as the crosslinking agent between the molecular skeletons. The characterization of the microspheres showed that the average particle size of the nanomicrospheres was 336 nm and that these microspheres maintained good stability at 278 °C. Meanwhile, the nanomicrospheres had good salt and temperature resistance and were able to maintain excellent swelling performance at a mineralization level of <10,000 mg/L and a temperature of <90 °C.

The experiment on the blocking property of the polymer microspheres shows that the nanomicrospheres used in the artificial core simulation drive have a good core blocking ability. The concentration of nanomicrospheres and the core permeability affect the blocking rate and resistance coefficient. Under the condition of certain permeability, the blocking rate and drag coefficient gradually increase with increasing polymer microsphere concentration. Conversely, under the condition of a certain concentration of polymer microspheres, the blocking rate and drag coefficient gradually decrease with increasing core permeability [36,37]. The experimental results indicate that PER nanomicrospheres can be used in artificial core simulation to simulate driving with good oil driving and recovery ability. It can effectively block the dominant channel of water flow in the rock core, and it can also deepen along the pore roar channel to achieve the role of deep driving. The experimental results indicated that compared with AM microspheres (without REQ modification), PER nanomicrospheres have a more significant effect on recovery enhancement operation, and the final recovery rate increased by 23.53% compared with the water drive stage. Thus, the PER nanomicrosphere blocking technology can considerably enhance oil recovery from reservoirs, offering a new solution for efficient and economic oil extraction.

Consequently, the improved performance and application potential of PER nanomicrospheres provides an innovative solution for the development and application of surfactant-modified polyacrylamide microspheres containing surfactants.

## Figures and Tables

**Table 1:** Experimental data table of drag coefficient, residual drag coefficient and blocking rate under different core permeability.

Permeability (×10^−3^ µm^2^).	Pressure Measurement Interval	Resistance Coefficient, *R*_f_	Residual Resistance Coefficient, *R*_ff_	Blocking Rate (%)
800	P0–P1	4.51	7.82	86.07
	P1–P2			
	P2–P3			
	P3–P4			
2000	P0–P1	3.47	6.01	79.13
	P1–P2			
	P2–P3			
	P3–P4			
4000	P0–P1	2.55	4.53	68.21
	P1–P2			
	P2–P3			
	P3–P4			

**Table 2:** Experimental data table of drag coefficient, residual drag coefficient, and clogging rate under different PER microsphere concentrations.

Concentration (mg/L)	Pressure Measurement Interval	Resistance Coefficient, *R*_f_	Residual Resistance Coefficient, *R*_ff_	Blocking Rate (%)
2000	P0–P1	4.33	8.70	83.03
	P1–P2			
	P2–P3			
	P3–P4			
3000	P0–P1	5.60	8.23	86.77
	P1–P2			
	P2–P3			
	P3–P4			
4000	P0–P1	5.71	9.75	90.32
	P1–P2			
	P2–P3			
	P3–P4			

## Data Availability

The data that support the findings of this study are available from the corresponding author, [X.L.], upon reasonable request.
